# The efficacy of electroacupuncture combined with Micro-Needle-Knife for allergic rhinitis: study protocol for a randomized controlled trial

**DOI:** 10.3389/fmed.2026.1756690

**Published:** 2026-03-13

**Authors:** Pengfei Qiu, Xue Wu, Ning Ye, Xiaofang Zhou, Jianfang Zhu, Xiayang Zeng

**Affiliations:** 1 Department of Acupuncture and Moxibustion, Zhejiang Hospital, Hangzhou City, Zhejiang, China; 2 The Second School of Clinical Medicine, Zhejiang Chinese Medical University, Hangzhou City, Zhejiang, China; 3 Department of Laboratory Medicine, Zhejiang Hospital, Hangzhou City, Zhejiang, China; 4 Department of Otolaryngology, Zhejiang Hospital, Hangzhou City, Zhejiang, China; 5 Department of Tui Na, Zhejiang Hospital, Hangzhou City, Zhejiang, China

**Keywords:** allergic rhinitis, electroacupuncture, Micro-Needle-Knife, protocol, randomized controlled trial

## Abstract

**Introduction:**

Allergic rhinitis (AR), classified as a hypersensitivity disorder profoundly impairing human quality of life, presents substantial clinical complexities. Among these, the management of moderate-to-severe AR cases constitutes a particularly challenging clinical scenario, which seriously affects their daily life, study, sleep, and mood. The efficacy of conventional treatments for AR remains suboptimal, necessitating an urgent exploration of alternative approaches to identify simpler, more convenient, effective, and cost-efficient therapeutic options for clinical AR management. This trial is designed to rigorously assess the efficacy and safety of electroacupuncture (EA) combined with Micro-Needle-Knife therapy as a novel therapeutic modality for patients with moderate-to-severe allergic rhinitis.

**Methods and analysis:**

This study protocol is a randomized controlled, patient-assessor-blinded trial. The trial will have a treatment period of 4 weeks and a follow-up period of 3 months. 90 eligible participants will be randomly assigned to the EA combined with Micro-Needle-Knife group and the drug group in a 1:1 ratio. The evaluation of all study parameters will be conducted across five distinct temporal phases: baseline assessment (week 0), two consecutive intervention phases (weeks 2 and 4), and two subsequent follow-up intervals (weeks 8 and 16). The primary outcome is the Total Nasal Symptom Score (TNSS). Secondary outcomes include Total Non-nasal Symptom Score (TNNSS), Rhinitis Quality of Life Questionnaire (RQLQ), serum chemokine Eotaxin, Inter Cellular Adhesion Molecule-1 (ICAM-1), and Eosinophil Cationic Protein (ECP). All adverse events will be evaluated during the trial.

**Conclusions:**

This study will preliminarily evaluate if EA combined with Micro-Needle-Knife is effective and safe in the treatment of AR.

**Clinical trial registration:**

https://clinicaltrials.gov/study/NCT06890260; identifier: NCT06890260.

## Introduction

Allergic rhinitis (AR), mediated by IgE, is a symptomatic nasal condition triggered by an immune response to allergen exposure (1). As a prevalent global health issue, statistics indicate that the global incidence of AR ranges from 10% to 25%, with a consistent upward trend (2). Preliminary studies conducted among urban populations in China reveal an average self-reported prevalence rate of 11.1% (3). Although AR is classified as a non-fatal inflammatory condition, its clinical implications extend beyond nasal symptoms to encompass a spectrum of systemic comorbidities. These include bronchial asthma exacerbation, chronic rhinosinusitis with potential nasal polyposis, eustachian tube dysfunction-mediated otitis media, and concurrent allergic conjunctivitis, thereby impacting quality of life and work productivity (4, 5).

The Working Group on “Allergic Rhinitis and its Impact on Asthma (ARIA)” of the World Health Organization (WHO) categorizes allergic rhinitis into mild and moderate-to-severe categories based on the severity of symptoms and their impact on quality of life. Patients experiencing symptoms that interfere with daily activities, studies, normal sleep, or other troubling conditions are diagnosed with moderate-to-severe allergic rhinitis (1). Moderate-to-severe allergic rhinitis is characterized by recurrent episodes, a prolonged course, and a significant impact on patients' quality of life. Consequently, this project will focus on conducting research on patients with moderate-to-severe allergic rhinitis.

The current strategies for treating allergic rhinitis (AR) encompass avoiding exposure to allergens, pharmacological treatment, immunotherapy, and patient education (6). For moderate to severe cases, the primary objective is to manage symptoms. The latest ARIA guidelines from 2016 strongly recommend a regimen combining short-term oral antihistamines with topical nasal corticosteroids for treatment (1). Despite the existing favorable therapeutic outcomes, Western medicine has yet to offer a definitive cure for allergic rhinitis. To alleviate clinical symptoms, long-term medication is inevitable, which not only imposes an economic burden on patients but may also lead to varying degrees of adverse effects, including nasal dryness, bleeding, and arrhythmia. Many patients are concerned about the adverse reactions of medications, making it challenging for them to adhere to standardized treatment protocols. Additionally, a significant portion of patients do not respond well to medications. Surveys (7–9) reveal that 60% of patients remain “highly interested” in exploring new therapies or medications, while 25% are continually experimenting with different drugs. Hence, there is a pressing need to identify practical, economical, safe, and efficacious alternative treatment options.

Many patients have started to explore traditional alternative therapies, with 17.5% of them willing to try acupuncture to alleviate rhinitis symptoms (10, 11). Despite studies (12–19) suggesting that there is currently insufficient evidence to either support or refute acupuncture's efficacy in treating allergic rhinitis, numerous patients still opt for acupuncture therapy due to its ability to relieve nasal symptoms, enhance quality of life, and offer a safe treatment option without toxic side effects, while also reducing drug dependence to some degree. EA and Micro-Needle-Knife therapy represent an emerging blend of traditional and innovative treatment methods within the acupuncture system. Prior research (20) has demonstrated that, compared to pharmacological treatment, simple EA at the Yingxiang point can improve symptoms of AR, including nasal congestion, nasal itching, sneezing, rhinorrhea, and nasal mucosa edema. Additionally, researchers (21, 22) have achieved comparable therapeutic outcomes to drug controls by utilizing conventional acupuncture combined with Micro-Needle-Knife therapy in the treatment of AR.

Previous research conducted by our team (23, 24) has likewise demonstrated that acupuncture alone or acupuncture combined with cupping therapy is effective in treating AR, yielding results comparable to standard pharmacological treatments. However, during our research, we observed that acupuncture alone involves numerous acupuncture points, has a relatively slow onset of action, a prolonged treatment duration, and can cause significant discomfort. Consequently, some patients are unable to adhere to the treatment due to their fear of acupuncture. While acupuncture combined with cupping therapy exhibits notable efficacy, the time commitment for a single treatment session is considerable. To enhance treatment efficacy and patient adherence, the author has consistently experimented with combining EA and Micro-Needle-Knife therapy in clinical practice. Given its ease of administration, minimal acupuncture point selection, reduced pain, rapid onset of action, high patient acceptance, and remarkable therapeutic outcomes, the combination of EA and Micro-Needle-Knife therapy stands out as an excellent option for treating AR. Nevertheless, to date, no research has investigated the efficacy of this combined treatment approach, leaving a lack of robust evidence to support its use.

An increasing number of clinical studies have confirmed the efficacy of acupuncture and moxibustion in treating AR (17–19). Currently, research into the mechanisms of acupuncture and moxibustion for AR has been conducted, focusing on interleukin-4 (IL-4), interleukin-6 (IL-6), interleukin-10 (IL-10), and IgE levels (25, 26). Previous research has demonstrated that acupuncture and moxibustion can decrease the EOS count in nasal secretions of AR patients (27, 28); however, there have been few studies reporting on the regulation of Eotaxin, ICAM-1, and ECP, which are closely associated with the chemotaxis, aggregation, and activation of EOS.

Therefore, this study focuses on patients with allergic rhinitis, utilizing both domestically and internationally recognized evaluation indicators to ascertain the clinical efficacy of EA combined with Micro-Needle-Knife therapy in treating AR, investigate the impact of this combined therapy on serum levels of Eotaxin, ICAM-1, and ECP in patients with moderate to severe AR and explore the potential mechanisms of immune regulation, thereby providing a basis for the clinical promotion of acupuncture and moxibustion in AR treatment, and ensure strict adherence to the Standard Operating Procedure (SOP) during implementation, aiming to provide high-quality evidence-based medical support for acupuncture treatment of AR.

## Method and analysis

### Study design

**Figure 1:**
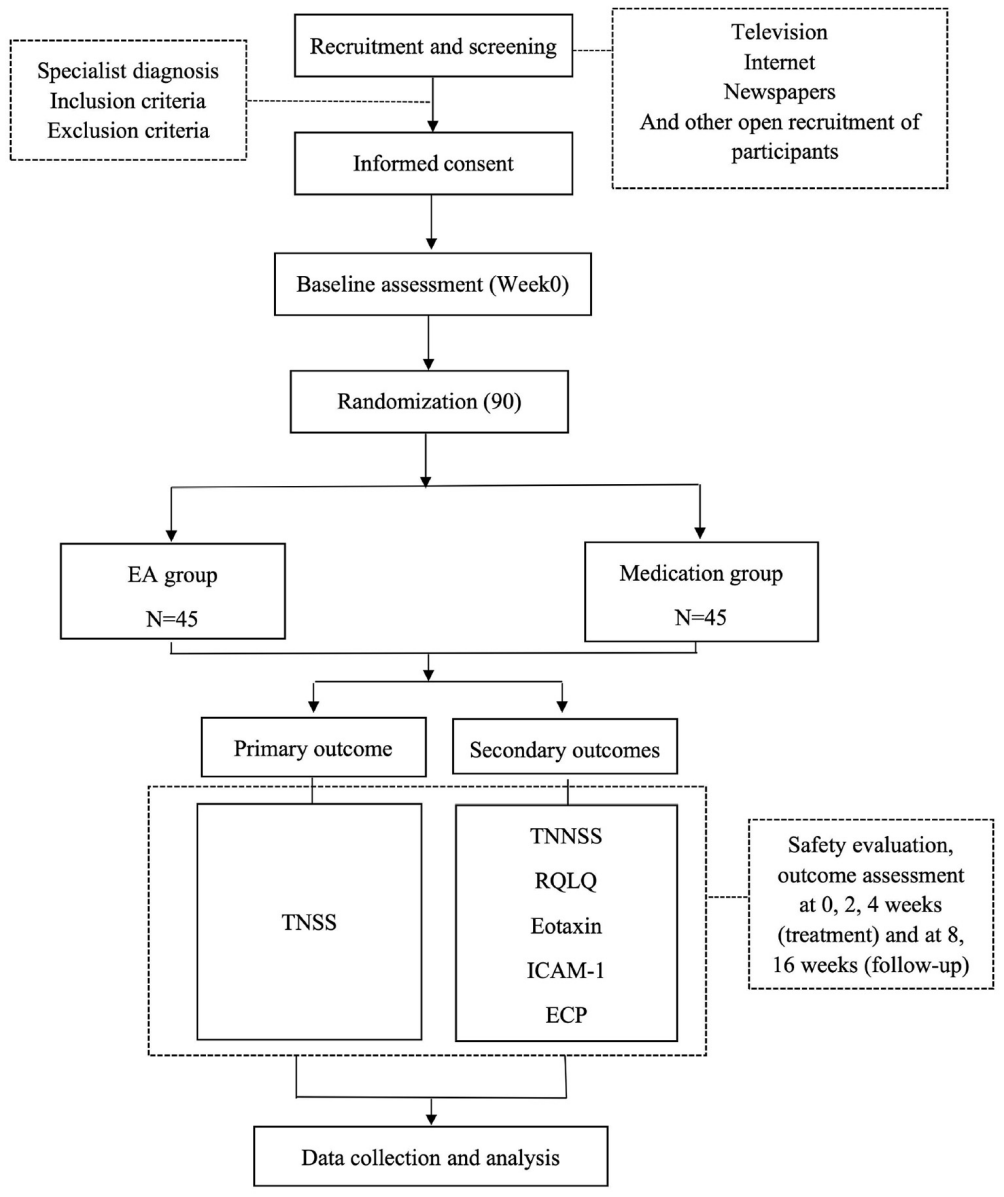
Flow chart of the study process. EA, electroacupuncture; TNSS, Total Nasal Symptom Score; TNNSS, Total Non-nasal Symptom Score; RQLQ, Rhinitis Quality of Life Questionnaire; ICAM-1, Inter Cellular Adhesion Molecule-1; ECP, Eosinophil Cationic Protein.

This study employs a randomized, parallel-arm, assessor-blinded controlled clinical trial design with dual masking of participants and outcome evaluators. Eligible individuals diagnosed with moderate-to-severe allergic rhinitis who meet predefined inclusion/exclusion criteria will be systematically allocated to either the EA intervention arm or conventional pharmacotherapy control arm. Efficacy assessment will be conducted at five distinct timepoints: baseline assessment (week 0), two consecutive intervention phases (weeks 2 and 4), and two subsequent follow-up intervals (weeks 8 and 16). The experimental framework is visually encapsulated in Figure 1, while the detailed chronological sequence of recruitment, intervention delivery, and outcome measurement is delineated in Table 1. Adherence to methodological transparency is ensured through compliance with the Standards for Reporting Interventions in Clinical Trials of Acupuncture (STRICTA) (29) checklist and Standard Protocol Items: Recommendations for Interventional Trials (SPIRIT) (30) guidelines, thereby guaranteeing comprehensive documentation of procedural integrity and reporting rigor throughout the study lifecycle.

**Table 1:** Schedule of enrolment, treatments, and assessments.

**Study period**	**Enrolment/Baseline**	**Treatment period**		**Follow-up period**	
**Assessment Point**	**1**	**2**	**3**	**4**	**5**
**Time**	−**2 Weeks to 0**	**2 Weeks** ±**3 Days**	**4 Weeks** ±**3 Days**	**8 Weeks** ±**3 Days**	**16 Weeks** ±**3 Days**
Eligibility screening	√				
Demographic data	√				
Case data	√				
Inclusion criteria	√				
Exclusion criteria	√				
Informed consent	√				
Treatment		√	√		
**Outcome Assessment**					
(1) TNSS	√	√	√	√	√
(2) TNNSS	√	√	√	√	√
(3) RQLQ	√	√	√	√	√
(4) Eotaxin	√		√		
(5) ICAM-1	√		√		
(6) ECP	√		√		
Safety assessment (EA)		√	√		
Safety assessment (Medication)		√	√		
Adverse events	√				

EA, electroacupuncture; TNSS, Total Nasal Symptom Score; TNNSS, Total Non-nasal Symptom Score; RQLQ, Rhinitis Quality of Life Questionnaire; ICAM-1, Inter Cellular Adhesion Molecule-1; ECP, Eosinophil Cationic Protein.

### Participant enrollment

The principal recruitment of study participants originates from Zhejiang Hospital. Recruitment strategies employ a multifaceted approach: researchers prioritize systematic follow-ups in specialized outpatient clinics and routine inpatient screenings. For eligible individuals, they meticulously log comprehensive details including full names, chronological ages, contact numbers, clinical diagnoses, and scheduled follow-up intervals, while fostering consistent bidirectional communication. To augment inclusivity, periodic outreach campaigns utilize conventional print publications and digital platforms such as WeChat, extending recruitment visibility to the general population. This dual-pronged methodology—combining targeted clinical engagement with community-wide dissemination—ensures both depth in participant selection and breadth in societal representation, thereby optimizing study generalizability while adhering to ethical recruitment protocols.

### Eligibility criteria

#### Diagnostic criteria

Diagnosis follows the criteria outlined in the “Guidelines for the Diagnosis and Treatment of Allergic Rhinitis (2022 Revised Edition)” (31) established by the Rhinology Working Group of the Otolaryngology-Head and Neck Surgery Branch of the Chinese Medical Association. Specific criteria are as follows:

(1) Clinical Symptoms: Presence of two or more symptoms (including two) such as sneezing, watery nasal discharge, nasal itching, and nasal congestion, with daily symptom duration or cumulative duration exceeding 1 h. May be accompanied by ocular symptoms including eye itching and conjunctival hyperemia;

(2) Physical signs: Commonly observed pale and edematous nasal mucosa with serous nasal discharge;

(3) Positive serum-specific IgE test. A definitive diagnosis of allergic rhinitis requires clinical manifestations consistent with serum-specific IgE test results.

#### Inclusion criteria

1. Patients with moderate to severe allergic rhinitis diagnosed by clinical and laboratory diagnostics;
2. Eligible participants must be adults aged 18–75 years inclusive, with no restrictions based on biological sex or national origin;
3. No antihistamines or nasal steroids were taken within 1 month before enrollment;
4. Those who voluntarily participate in the study and sign the informed consent, and can adhere to the outpatient treatment for 4 weeks.

#### Exclusion criteria

1. Atrophic rhinitis, vasomotor rhinitis, hypertrophic rhinitis, nasal polyps, acute and chronic sinusitis, eosinophilia, severe deviation of nasal septum, non allergic rhinitis;
2. Asthma, urticaria and other allergic diseases, nasal congestion, runny nose and sneezing caused by a cold;
3. Patients with severe diseases such as cardiovascular, cerebrovascular, liver, kidney, and hematopoietic system;
4. Pregnant or lactating women, psychiatric patients, patients with malignant tumors.
5. Received immunotherapy within the past year.

#### Discontinuation criteria

1. Participants experiencing severe adverse events, significant physiological alterations, or unanticipated incidents during the study, rendering them unfit for ongoing involvement;
2. Critical complications or health status decline necessitating urgent intervention during the study;
3. Subjects are uncooperative and noncompliant with treatment, and repeated explanations by clinicians do not work;
4. Serious adverse events due to needling during the study, such as serious infection, coma, shock, death, etc., should be immediately reported to the Principal Investigator and the trial should be immediately discontinued.

#### Elimination criteria

Cases that have been enrolled but have failed to complete treatment and follow-up in accordance with the established study protocol shall be considered to be discharged as indicated below:

1. Withdrawal of informed consent after grouping for personal reasons;
2. Withdrawal of cases from the trial if an adverse event occurs after inclusion and serious complications require suspension of the trial;
3. Cases that do not complete the entire course of treatment and affect the final judgment of efficacy are treated as dropouts.

### Randomization and allocation concealment

A sealed-envelope randomization approach is employed for participant allocation. Individuals meeting both inclusion and exclusion criteria are formally enrolled, after which randomization staff or clinical investigators assign them using pre-prepared allocation envelopes. The randomization protocol is generated by the Clinical Evaluation Center at the participating institution, with the designated generator explicitly excluded from subsequent statistical analysis of this project. The complete randomization framework, including both the allocation scheme and its generation parameters, constitutes the “blind code,” which is sealed, signed by the scheme generator, and safeguarded by a specialized administrator external to the study team. Subgroup participants remain uninvolved in statistical data analysis. Efficacy endpoint assessments are exclusively managed by blinded evaluators unaware of subgroup assignments, adhering to a triple-separation principle that strictly isolates the roles of primary investigators, acupuncture practitioners, and statistical analysts. This structure ensures methodological rigor while maintaining blinding integrity throughout the evaluation process.

### Blinding

Due to the specificity of the treatment program of this subject, the needling operator must be in direct contact with the subject, and it is not possible to blind him/her, so single blinding is implemented. However, the patient, the evaluator of observational indicators and the statistician are blinded. At the same time to realize the separation of the randomizer, the therapist and the evaluator of observational indicators. The final data shall be counted by a person who is not aware of the specifics of the experimental design, and the subjects shall be blinded to the effect of the trial.

The blinding protocol in this trial was not fully implemented, failing to achieve complete blinding. The practitioners were unable to maintain blinding, which introduced potential bias into the results. In future clinical research reports, we will identify potential sources of bias in blinding, propose evaluation methods, and assess their impact on outcomes. We will objectively examine the impact of bias on the strength and credibility of study conclusions, as well as its limitations on the applicability and generalizability of findings. Additionally, post-treatment questionnaires will be administered to patients to assess their treatment expectations, thereby quantifying the success of the blinded design implementation.

### Intervention

Participants will be assigned to receive either EA integrated with Micro-Needle-Knife therapy or conventional pharmacological intervention. Both treatment arms will follow an identical 4-week active intervention phase, immediately succeeded by a 12-week observational follow-up phase to monitor long-term outcomes.

### EA combined with Micro-Needle-Knife

#### EA operation

The acupuncture points of Yingxiang (LI20) and Shangyingxiang (EX-HN8) are taken with reference to the 2006 National Standard of the People's Republic of China (GB/T 12346-2006) “Acupoint Names and Localization.” The patient takes the supine position, the local disinfection of acupuncture points, using 0.22 mm × 25 mm Le Moxibustion brand disposable sterile acupuncture needles (Suzhou Acupuncture and Moxibustion Supplies Co., Ltd.), needling bilateral LI20 and EX-HN8 points, the tip of the needle toward the root of the nose diagonal stabbing 5–10 mm, in order to the degree of soreness and swelling sensation of the nose, the line of flat tonic and flat diarrhea method. After obtaining qi, Yingdi KWD-808I Pulse Acupuncture and Moxibustion Therapeutic Instrument is used, and one end of the output electrode is clamped on the LI20 and EX-HN8 points on the same side respectively, and the waveform of the EA waveform is selected to be sparse and dense wave with the frequency of 2/100 Hz, and the intensity of the current is tolerated by the patient to the extent of the current, and the time of electrification is 30 min, and the therapy is carried out once in every other day, and three times in every week, for 4 weeks in total.

#### Micro-Needle-Knife operation

**Figure 2:**
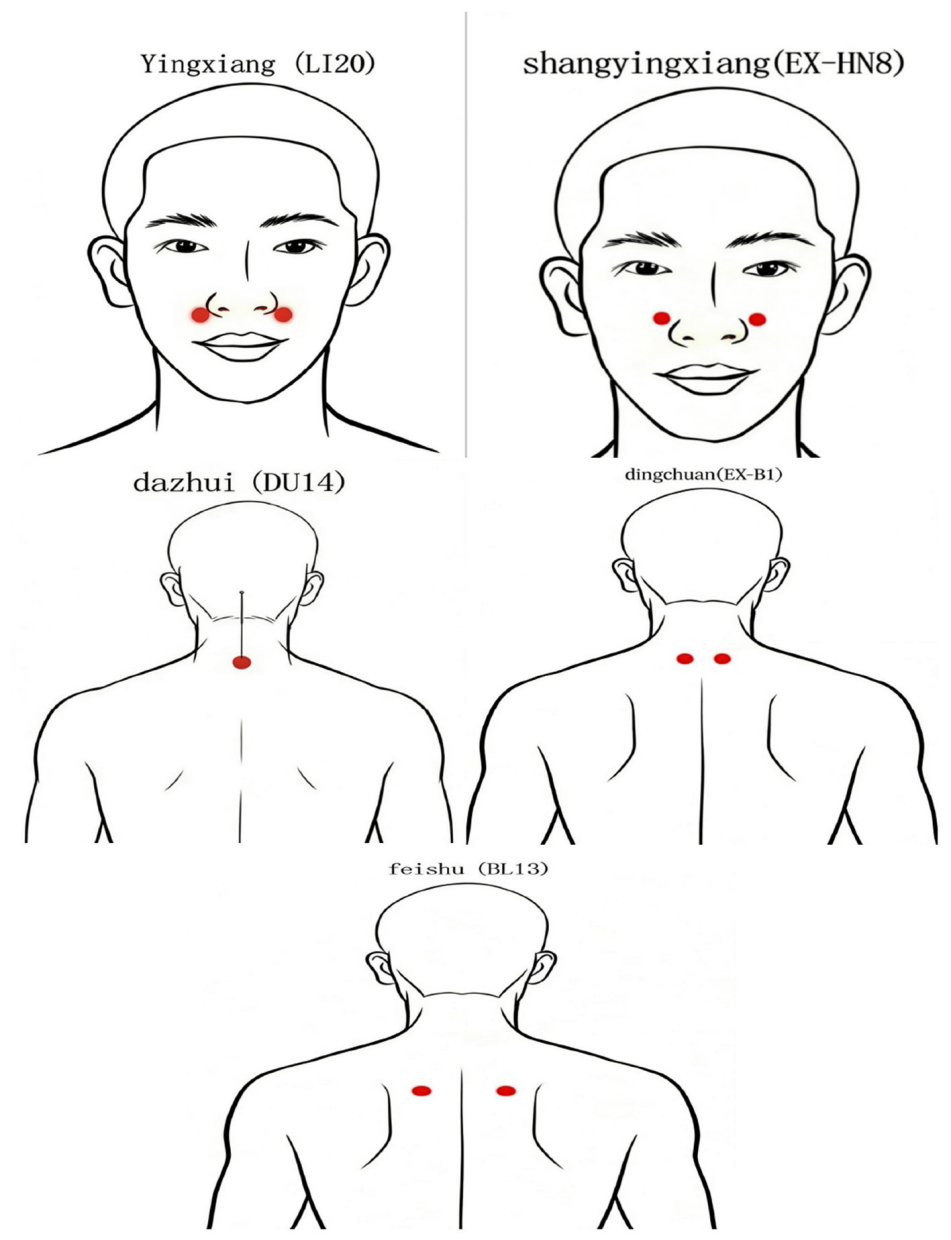
Acupoint annotation diagram.

Fixed points: Treatment points at the occipital-cervical segment: A Shi acupoints, which are referred to hard knots or pressure points at the suboccipital line, the tip of the transverse process of the 1st cervical vertebra, and the paracentral points of the 2nd and 3rd cervical vertebrae. Treatment points at the cervicothoracic junction: Dazhui acupoint (DU14), Dingchuan acupoint (EX-B1) (bilaterally) and Feishu acupoint (BL13) (bilaterally). The Patient takes a seated position, head down, placed on the treatment pillow, select the obvious pressure point for marking, routine skin disinfection with 0.35 mm × 25 mm Le Moxibustion brand Micro-Needle-Knife (Suzhou Acupuncture and Moxibustion Products Co., Ltd.) straight stabbing 0.5–1.5 cm, cutting and loosening the needle after the line, with sterile gauze to stop the bleeding by pressing on the needle eye, weekly treatment once, a total of 4 weeks of treatment. Table 2 succinctly summarizes the acupuncture point positions utilized in the experimental trial. Figure 2 shows the locations of each acupoint.

**Table 2:** Indication and localization of acupoints for the management of AR.

**Acupoints**	**Location**
A Shi acupoints	A Shi points refer to the hard knots or pressure points at the suboccipital line, the tip of the transverse process of the 1st cervical vertebra, and the paracentral points of the 2nd and 3rd cervical vertebrae.
Ying Xiang (LI20, bilateral)	Ying Xiang is near the midpoint of the outer edge of the nasal wing, when in the nasolabial groove.
Shang Ying Xiang (EX-HN8, bilateral)	Shang Ying Xiang belongs to extra meridian acupoint. It is on the face, at the junction of alar cartilage and turbinate, near the upper end of nasolabial groove.
Da Zhui (DU14)	Da Zhui is located in the posterior midline, and in the depression under the spinous process of the seventh cervical vertebra.
Ding Chuan (EX-B1, bilateral)	Ding Chuan is on the back, 0.5 inches below the spinous process of the seventh cervical vertebrae.
Fei Shu (BL13, bilateral)	Fei Shu is on the back, 0.5 inches below the spinous process of the third thoracic vertebrae.

### Medication group

The participants in this group will be given cetirizine hydrochloride tablets (10 mg ^*^ 24 tablets, Suzhou Sinochem Pharmaceutical Industry Co., Ltd., China National Pharmaceutical License: H20030447), 1 tablet per day, and budesonide spray (64 μg ^*^ 120 sprays, AstraZeneca Pharmaceuticals Co., Ltd., China National Pharmaceutical License: J20090079). Dosage: The starting dose is 2 sprays in each nostril twice daily, after 3 days, it is reduced to 1 spray in each nostril twice daily, and then changed to 1 spray once daily after 1 week, which is used as the maintenance dose. The dosage will be increased or decreased at the patient's discretion according to the symptomatic condition during the course of treatment for a total of 4 weeks of treatment and observation. No other drugs for allergic rhinitis will be used during the treatment period.

### Outcome measures

#### Primary outcome measure

The primary outcome measure is the TNSS, a standardized tool used to quantitatively assess the severity of nasal symptoms (nasal congestion, itchy nose, runny nose, sneezing) in patients with rhinitis, with each symptom scored on a scale of 0–3 and a total score of 0–12, with higher scores denoting greater symptom severity. The advantages of this tool are its simplicity, standardization and comprehensiveness, and it is commonly used in clinical diagnosis, efficacy assessment and scientific research (32–34), but it needs to be combined with other assessment tools and pay attention to the subjective differences of patients.

#### Secondary outcome measures

Secondary outcome measures included the TNNSS, RQLQ, serum chemokine Eotaxin, ICAM-1, and ECP. The TNNSS (35, 36) serves as a metric to evaluate non-nasal symptom severity in patients with rhinitis. It covers five symptoms: runny nose, tearing, ocular itchiness or nasal, oral maxillary pain or nasal, and headache, each of which is rated on a scale of 0–3, and the total score is the sum of the scores of each symptom, with higher scores resulting in worse symptoms, and is often used in conjunction with the TNSS for a comprehensive assessment of the condition. The RQLQ scale (37, 38) covers a number of dimensions, including sleep problems, activity limitation, ocular symptoms, nasal symptoms, emotional distress, practical problems, and non-nasal/ocular symptoms, and quantifies the impaired quality of life by the patient's rating of the degree of distress (0 to 6) for each dimension. Higher cumulative scores reflect stronger rhinitis-related quality-of-life impairment. Eotaxin is the most potent of the eosinophil chemokines identified and plays an important role in the pathology of allergic rhinitis (39). ICAM-1 is a member of the immunoglobulin superfamily of adhesion molecules, which causes the development of allergic reactions and can lead to the difficulty of curing allergic rhinitis (40, 41). ECP is a toxic protein released by activated eosinophils which plays an important role in the occurrence and development of allergic rhinitis (42).

### Safety evaluation

The acupuncturist is responsible for recording the safety parameters throughout treatment/follow-up and promptly record them on the Adverse Event Log.

(1) Evaluation of the safety of EA and Micro-Needle-Knife: Dizziness, missed needles, broken needles, intolerable needle pain, local infection, hematoma, etc.; other post-needle discomfort (defined as post-needle pain, palpitations, dizziness, headache, etc. of ≥ 1 h's duration after needling) symptoms, average duration, and degree.

(2) Adverse drug reactions: drowsiness, somnolence, headache, dizziness, dry mouth, and gastrointestinal discomfort.

### Quality control and data management

The study team will establish standardized operating protocols (SOPs) to govern all trial procedures. A comprehensive training program will be conducted 1 month prior to the formal initiation of the clinical trial, specifically designed to equip all participating investigators with standardized operational knowledge. The training curriculum emphasizes mastery of the trial execution blueprint and strict adherence to protocol-defined SOPs, ensuring that each clinical investigator achieves full proficiency in research methodologies and implementation specifics—critical for safeguarding the scientific validity and authenticity of trial outcomes.

All clinical observations and recorded data undergo stringent validation through multi-tiered verification processes to guarantee data integrity. This meticulous approach ensures that every analytical conclusion and reported outcome originates exclusively from primary, uncorrupted source data, thereby preserving the trustworthiness of research conclusions. Professional data governance agencies are contracted to oversee clinical data management workflows, supplemented by monthly quality assurance audits to enforce compliance with research standards. For participants who withdraw at any stage, systematic documentation of withdrawal rationales is mandated, with subsequent statistical analysis of attrition rates to evaluate participant retention efficacy. This systematic framework integrates quality control mechanisms across training, data acquisition, and analysis phases, ensuring methodological rigor while maintaining transparency in reporting.

### Sample size estimation

Sample size estimation for non-inferiority of two independent samples is done through the website “Powerandsamplesize.com”. Based on previous literature (24), the TNSS scores are 4.08 ± 0.39 in the test group and 4.57 ± 0.72 in the control group after 8 weeks of treatment. Taking the test level of α = 0.05 (two-sided), test efficacy 1–β = 0.90, and the ratio of the two groups being 1:1, the sample size is calculated to obtain a sample size of at least 39 individuals in each of the two groups. Due to factors such as shedding during the test, the sample size needs to be expanded by 15%, and it is finally calculated that at least 45 subjects will be needed in each group, totaling 90 in the two groups.

### Statistical analysis

Statistical analysis will be performed using SPSS 25.0 software. All subjects included in the randomization will be analyzed for general conditions at baseline; subjects completing the last follow-up visit will be included in the original analysis. Depending on the characteristics of the data, continuous variables that conformed to normal or approximately normal distribution are expressed as mean (standard deviation) (Mean (SD), and continuous variables that don't conform to normal distribution are expressed as median (P25, P75). Measurement data are first tested for normality, and *t*-test, paired *t*-test, ANOVA, and analysis of covariance (ANCOVA) are used for those conforming to normal distribution, while rank-sum test is used for those not conforming to normal distribution. The count data are tested by chi-square test, Fisher's exact test, etc.; *P* < 0.05 is used to indicate that the difference is statistically significant.

### Ethical approval and study registration

Ethical clearance for this investigational study has been formally secured through the Zhejiang Hospital Institutional Review Board (Approval Code: ZJHIRB-023K). Prior to enrollment, prospective participants will receive comprehensive disclosures regarding the trial's primary objectives, anticipated therapeutic benefits, and associated procedural risks. Written informed consent, obtained pre-participation, serves as a mandatory prerequisite to ensure voluntary engagement, granting individuals full decisional autonomy regarding study involvement. To maintain participant confidentiality, the clinical case report forms will exclusively record demographic details such as age, while utilizing participant initials for non-identifiable reference. The research protocol has been prospectively registered in the Clinical Trials Registry with a unique identifier (NCT06890260), enabling traceable oversight throughout the study lifecycle. This ethical framework adheres to international standards, integrating rigorous participant protection measures with transparent reporting protocols to uphold scientific integrity and regulatory compliance.

## Discussion

AR, as an allergic condition that significantly impacts human life quality, poses numerous clinical challenges, particularly for patients with moderate to severe symptoms. Consequently, this project will concentrate its research efforts on patients suffering from moderate to severe allergic rhinitis. Currently, treatment primarily revolves around pharmacological interventions, which often come with significant side effects, making long-term adherence or complete cure difficult for patients. Hence, there exists an immediate imperative to investigate more streamlined, accessible, cost-effective, and clinically efficacious therapeutic strategies. “EA combined with Micro-Needle-Knife therapy for allergic rhinitis” possesses a certain foundation of clinical efficacy. However, it lacks support from systematic clinical research findings, hindering its widespread popularization and promotion. Consequently, a critical necessity emerges for conducting a randomized controlled trial of high methodological rigor to systematically examine the therapeutic effectiveness and safety profile of EA combined with Micro-Needle-Knife therapy in treating AR. To ascertain the therapeutic effect of this combined therapy in managing moderate to severe allergic rhinitis, this study utilizes the nasal application of glucocorticoids (budesonide nasal spray) in conjunction with oral antihistamines (cetirizine hydrochloride tablets), as recommended by the ARIA guidelines, as the comparator for efficacy evaluation.

Studies suggest that EA exhibits robust anti-inflammatory and anti-allergic effects in treating AR (43, 44). In the field of Micro-Needle-Knife medicine, it is widely acknowledged (21, 22, 45) that nasal autonomic nerve dysfunction plays a crucial role in the pathogenesis of rhinitis. Inflammation within the nasal cavity constitutes the primary pathological change in AR. By using Micro-Needle-Knife to release tension in neck soft tissue, alleviating traction and compression on the autonomic nerves, and restoring the balance of autonomic nerve conduction and metabolism, the nasal symptoms and quality of life of AR patients can ultimately be improved. However, further investigation is needed to uncover the immune mechanisms underlying EA and Micro-Needle-Knife therapy for AR (46).

First of all, an important pathological feature of AR is the chemotaxis, migration, and aggregation of eosinophils into the local nasal mucosa tissue through degranulation and the production and release of a series of inflammatory mediators, resulting in damage to the nasal mucosa and exacerbating the local inflammatory response (47). EOS is the main cell in the chronic inflammatory process of AR delayed phase, and the activation releases inflammatory mediators such as ECP, which prolongs the AR symptoms (48). Therefore, EOS is considered an important therapeutic target for the control of allergic diseases (49), and inhibition or antagonism of eosinophil chemotaxis and activation could potentially be a new strategy for the treatment of allergic diseases. And the local chemotaxis, aggregation and degranulation of EOS are closely related to the interaction with chemokines Eotaxin and ICAM-1 (50, 51). Therefore, we intend to explore the possible immunomodulatory mechanisms through the effects of EA combined with Micro-Needle-Knife in the treatment of moderate-to-severe AR on serum Eotaxin, ICAM-1, and ECP, and to provide a partial basis for the clinical promotion of acupuncture in the treatment of AR.

Secondly, we selected TNSS, TNNSS, and RQLQ as outcome measures for evaluating the efficacy of EA combined with Micro-Needle-Knife in treating moderate-to-severe allergic rhinitis, primarily due to their ability to provide a multidimensional, comprehensive, and objective assessment of treatment outcomes. TNSS (32–34) specifically quantifies the severity of core nasal symptoms (e.g., nasal congestion, rhinorrhea, sneezing, nasal pruritus), while TNNSS (35, 36) complements this by evaluating non-nasal related symptoms (e.g., ocular pruritus, throat discomfort, headache), thereby capturing the systemic impact of allergic rhinitis. RQLQ (37, 38), in turn, assesses the disease's impact on quality of life across dimensions such as sleep, daily activities, social interactions, and emotional wellbeing. The integration of these three metrics simultaneously measures both symptomatic improvement and enhancement in quality of life, aligning with the composite evaluation requirements of “symptoms-function-quality of life” in clinical research. And this approach can ensure the comprehensiveness and scientific rigor of efficacy evaluation.

Overall, our findings will contribute to today's international understanding of the clinical relevance and efficacy of acupuncture and AR, leading to the introduction of acupuncture as a potential treatment option for AR patients with minimal side effects, aiming to alleviate their rhinitis symptoms and improve their quality of life and work participation.

## Limitations

However, our study has some methodological limitations, as the subject is acupuncture treatment and the acupuncture operator has to be in direct contact with the patient, to whom blinding can't be applied. But blinding is implemented for all other participants (including patients, those who record and enter observational indicators, and data statisticians), and none of these participants are aware of the specific grouping of patients. Furthermore, patients are required to attend weekly clinical appointments for three acupuncture treatments, with each session exceeding 1 h in duration. This schedule significantly amplifies time investment and logistical demands, contrasting sharply with the convenience of home-based pharmacological administration. The cumulative time commitment—encompassing travel, waiting, and treatment duration—creates a substantial barrier to adherence when compared to the simplicity of self-administered oral medications at home. This disparity in convenience underscores the practical challenges of acupuncture-based interventions in routine clinical practice, particularly for individuals with time constraints or limited mobility.

## Conclusion

In summary, this study protocol is a randomized controlled, patient-evaluator-blinded trial. Its purpose is to explore the clinical efficacy and immunomodulatory mechanism of EA combined with Micro-Needle-Knife in the treatment of AR, and to form a standardized, effective, and easy-to-promote acupuncture treatment protocol for the treatment of AR. It provides a partial basis for the clinical promotion of acupuncture methods for the treatment of AR.
